# Percutaneous unilateral biportal endoscopic discectomy for symptomatic lumbar disc herniation in geriatric patients

**DOI:** 10.3389/fsurg.2024.1519952

**Published:** 2025-01-17

**Authors:** Rongqing Qin, Anhong Guan, Min Zhu, Pin Zhou, Bing Zhou, Ruihua Zhou, Zaiyong Guan

**Affiliations:** ^1^ Department of Spinal Surgery, Gaoyou People’s Hospital, Yangzhou, Jiangsu, China; ^2^ Department of Orthopedics, The Third Clinical Medical College of Yangzhou University, Yangzhou, Jiangsu, China; ^3^ Department of Medical Image, Gaoyou People’s Hospital, Yangzhou, Jiangsu, China; ^4^ Department of Orthopedics, Gaoyou Hospital of Integrated Traditional Chinese and Western Medicine, Yangzhou, Jiangsu, China

**Keywords:** unilateral biportal endoscopic discectomy, lumbar disc herniation, minimally invasive, geriatric, spine surgery

## Abstract

**Purpose:**

The purpose of this study is to investigate the surgical efficacy and safety of percutaneous unilateral biportal endoscopic discectomy (UBED) for symptomatic lumbar disc herniation (LDH) in geriatric patients.

**Methods:**

Seventy-two geriatric patients, aged 65–86 years (mean age: 73.2 years), with single or two-level LDH who underwent UBED from January 2020 to September 2022 were retrospectively analyzed. Clinical outcomes were evaluated based on operation time, total blood loss, hospital stay, visual analog scale (VAS) scores for leg pain, Oswestry disability index (ODI) scores, modified MacNab criteria, and postoperative magnetic resonance imaging findings.

**Results:**

Surgery was successfully performed on all geriatric patients, with a mean operation time of 46 min (range: 32–68 min). All patients were followed up for an average duration of 14.2 ± 1.9 months (range: 12–16 months). The leg pain VAS score decreased from 8.37 ± 1.21 preoperatively to 2.03 ± 0.61 immediately after surgery, 1.56 ± 0.32 at 1 month postoperatively, 1.16 ± 0.45 at 6 months postoperatively, and 0.91 ± 0.26 at 12 months postoperatively. Similarly, the ODI score also decreased from 61.21 ± 11.06 preoperatively to 27.52 ± 10.41 immediately after surgery, 19.12 ± 7.05 at 1 month postoperatively, 12.17 ± 5.21 at 6 months postoperatively, and 8.56 ± 4.32 at 12 months postoperatively. Statistically significant differences were observed in both VAS and ODI scores at each follow-up time point when compared with preoperative parameters (*P* < 0.01). Also, there were 53 excellent cases, 12 good cases, and 7 fair cases based on the modified MacNab criteria at 12 months postoperatively, resulting in an excellent and good rate of 90.2%. Only three cases were found to be complicated by low extremity numbness, all of which were recovered via conservative treatment in 3 weeks. No infections or iatrogenic neurological deficits occurred in all patients.

**Conclusions:**

We concluded that UBED achieved satisfactory results and provided a minimally invasive, effective, and safe alternative for the treatment of symptomatic LDH in geriatric patients.

## 1 Introduction

Symptomatic lumbar disc herniation (LDH) with low back pain and/or sciatica is a disturbing disease (1). The radicular syndrome affects millions of people worldwide (2). While conservative treatment generally yields satisfactory results, some studies also have reported that LDH can be naturally absorbed (3, 4). However, for patients who do not respond to a period of conservative treatment, surgery seems necessary (5). In 1934, Mixter and Barr first attempted posterior laminectomy to decompress nerve roots and resect herniated discs for treating patients with symptomatic LDH (6). On this basis, Caspar introduced microscopic discectomy in the 1970s (7). Afterward, open lumbar microdiscectomy (OLM) had been regarded as the standard surgical approach for symptomatic LDH (8). Since the late 1980s, Kambin et al. introduced endoscopic and arthroscopic equipment in lumbar decompressive surgery through a transforaminal approach (9, 10). Accompanied by improvements in optical equipment and surgical instruments, percutaneous endoscopic lumbar discectomy (PELD) has been widely implemented for LDH, lumbar spinal stenosis, and other spinal conditions (11–14). In recent years, the minimally invasive technique, named unilateral biportal endoscopic discectomy (UBED), has also gained popularity in the treatment of lumbar disc diseases (15–17). UBED is performed via two independent channels on the unilateral side: one for visualization and the other for working instruments. Compared with OLM, UBED reduces the destruction of paraspinal muscles while providing a clear and magnified surgical field of vision, which improves operational flexibility and helps the surgeon to conduct precise and complete decompression (16, 18, 19). Although UBED has achieved favorable results in treating LDH, the complications associated with this technique have been reported (20). At present, the number of geriatric patients with symptomatic LDH is increasing with the global aging population. However, few studies have explored the surgical efficacy of UBED in older adults. In the present study, we described the clinical efficacy and safety of UBED for geriatric patients with symptomatic LDH.

## 2 Materials and methods

### 2.1 Patient population

After obtaining approval from the Institutional Review Board, we included 72 geriatric patients (43 men and 29 women) with lower extremity radiculopathy caused by LDH who underwent UBED under general anesthesia in our spinal surgery department from January 2020 to September 2022. The patients’ ages ranged from 65 to 86 years (mean age: 73.2 years). The mean duration of clinical symptoms was 7.8 ± 4.1 months, ranging from 2 to 12 months. After being identified by lumbar flexion and extension x-rays, computed tomography (CT), and magnetic resonance imaging (MRI), all enrolled patients were diagnosed with single or two-level LDH, including 9 cases at L3–L4, 29 at L4–L5, 23 at L5–S1 level, and 11 involving both L4–L5 and L5–S1 levels. Among the 72 patients, 11 had internal medical comorbidities such as diabetes, hypertension, and coronary heart disease. All patients had a minimum follow-up of 1 year. The baseline characteristics of participants are presented in Table 1.

**Table 1:** Baseline characteristics of the included patients.

Patients (*N* = 72)			
Male/female, *n* (%)			43 (59.7)/29 (40.3)
Age (years)			73.2 ± 8.5 (range: 65–86)
BMI (kg/m^2^)			24.8 ± 2.7
Duration of symptoms (months)			7.8 ± 4.1 (range: 2–13)
Duration of follow-up (months)			14.2 ± 1.9 (range: 12–16)
Disc level, *n* (%) (*N* = 83)			
	Single level	Two level	
L3–L4	9	0	*n* = 9 (10.8)
L4–L5	29	11	*n* = 40 (48.2)
L5–S1	23	11	*n* = 34 (41.0)
Type of herniation, *n* (%) (*N* = 83)			
Central			*n* = 31 (37.3)
Paracentral			*n* = 52 (62.7)

### 2.2 Inclusive and exclusive criteria

Inclusion criteria included the following: (1) patients aged 65 years or older; (2) cardinal symptom of leg pain with or without back pain due to single or two-level LDH; (3) positive nerve root tension signs; (4) imaging findings from preoperative x-rays, CT scans, and MRI consistent with clinical symptoms; (5) failure of conservative therapy for at least 3 months; and (6) availability of sufficient clinical data and at least 1 year of follow-up. Exclusive criteria included the following: (1) foraminal or far-lateral disc herniation; (2) lumbar instability or spondylolisthesis confirmed by lumbar flexion and extension x-rays; (3) presence of infected lesions along the puncture path; (4) history of lumbar spine surgery; and (5) serious physical illnesses, mental disorders, or abnormal blood coagulation function.

### 2.3 Surgical procedures

All operations were performed under general anesthesia with the patient in the prone position, with the hips and knees flexed. The target intervertebral space was confirmed via C-arm fluoroscopy. Two skin incisions centered around the target intervertebral space were made on the unilateral side. The endoscope and surgical instruments were introduced through the observational and working channels, respectively. Initially, the soft tissue in the surgical field of vision was cleared using radiofrequency ablation and forceps to create a working space. Then, the junction between the spinous process and vertebral plate at the target intervertebral space was identified. The inferior lamina of the upper lumbar vertebral body and the superior lamina of the lower lumbar spine were partially removed using abrasive drills and vertebral plate rongeur. The ligamentum flavum was carefully dissected and removed using Kerrison punches and a radiofrequency probe. After identifying the dural sac and nerve root, the annulus of the protruding intervertebral disc was dissected and exposed. Bleeding was carefully stopped, and discectomy was then performed using nucleus forceps. A 90° hooked probe was used to check for any residual fragments. The pulsation of the dural sac and the decompression of the nerve root were confirmed finally. After meticulously arresting bleeding again, a rubber drainage tube was placed outside the lamina, and the surgical incision was sutured.

### 2.4 Efficacy evaluation

Clinical efficacy was primarily evaluated using leg pain visual analog scale (VAS) scores and Oswestry disability index (ODI) scores immediately after surgery and at 1, 6, and 12 months postoperatively. Patient satisfaction was evaluated using the modified MacNab criteria (excellent, good, fair, and poor). Additional clinical evaluations included operation time, postoperative MRI findings, complications with subsequent remedies, recurrence of symptoms, time spent on bed rest, and duration of hospitalization.

### 2.5 Statistical analysis

Statistical analysis was conducted using SAS software (version 9.4). Data were presented as mean ± standard deviation. The one-way ANOVA followed by Dunnett’s *t*-test was used to compare leg pain VAS and ODI scores, respectively. *P* < 0.05 was considered statistically significant.

## 3 Results

**Figure 1:**
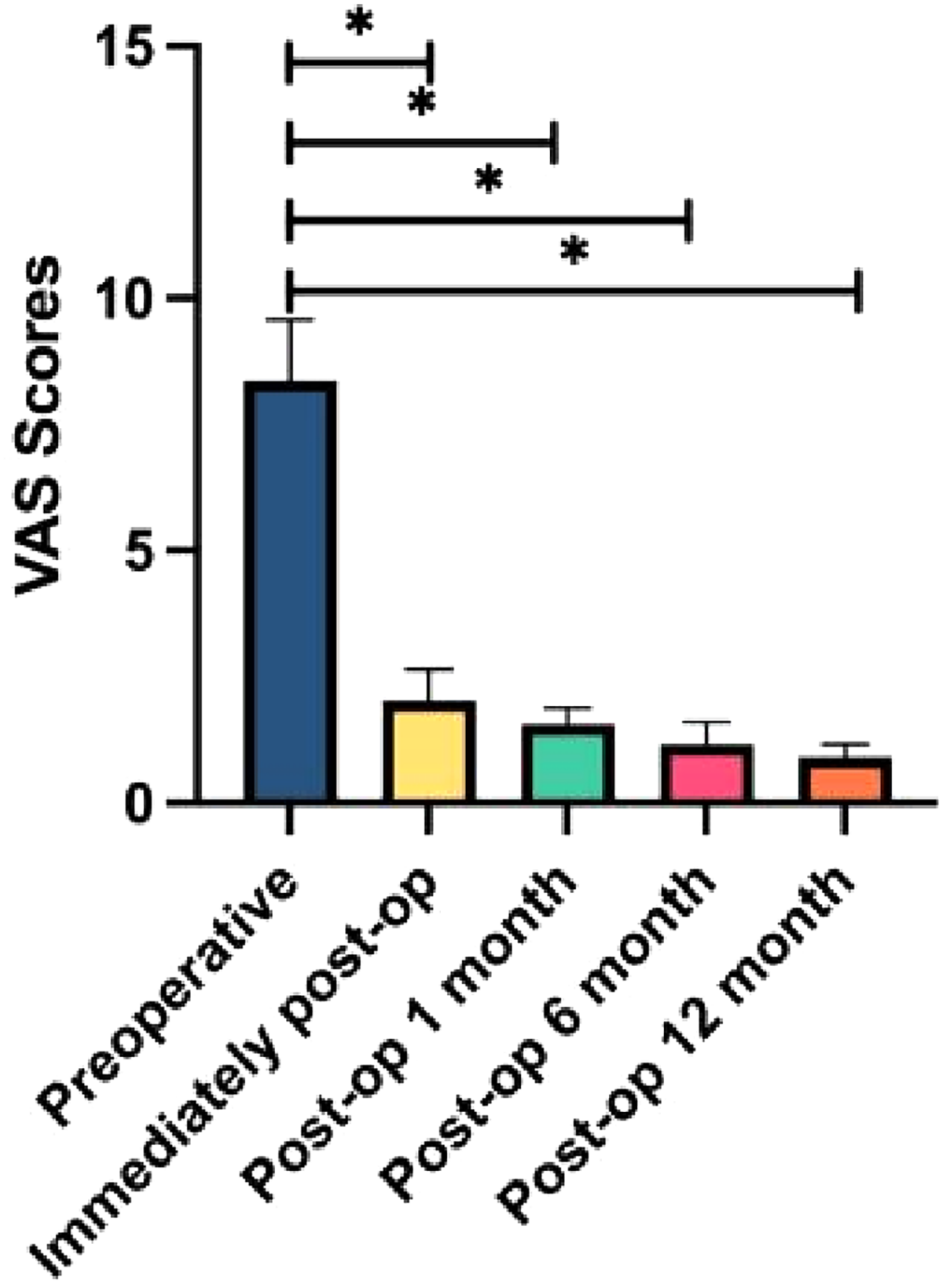
Histograms for leg pain VAS scores (*n* = 72). *P < 0.01.

**Figure 2:**
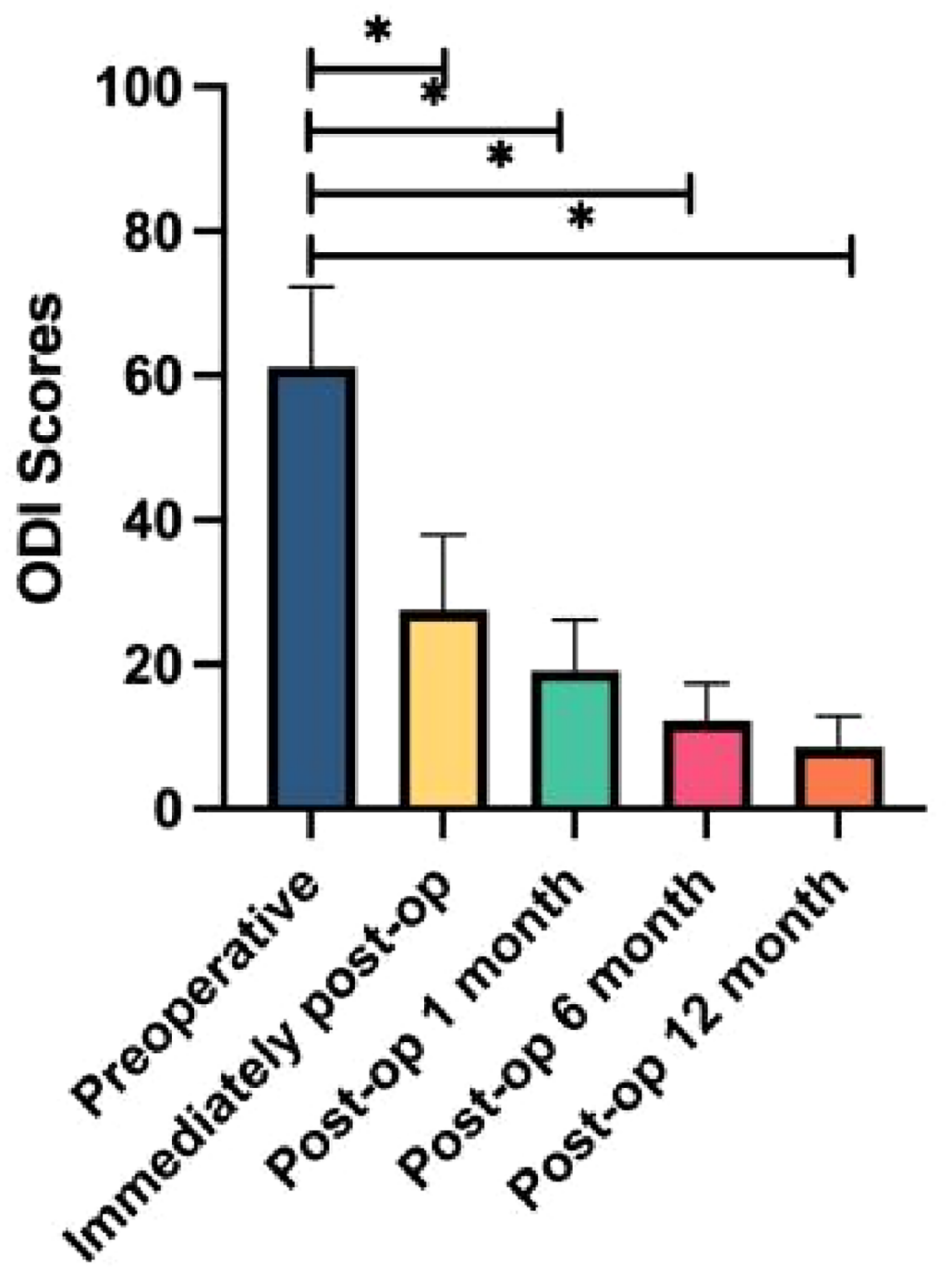
Histograms for ODI scores (*n* = 72). *P < 0.01.

**Figure 3:**
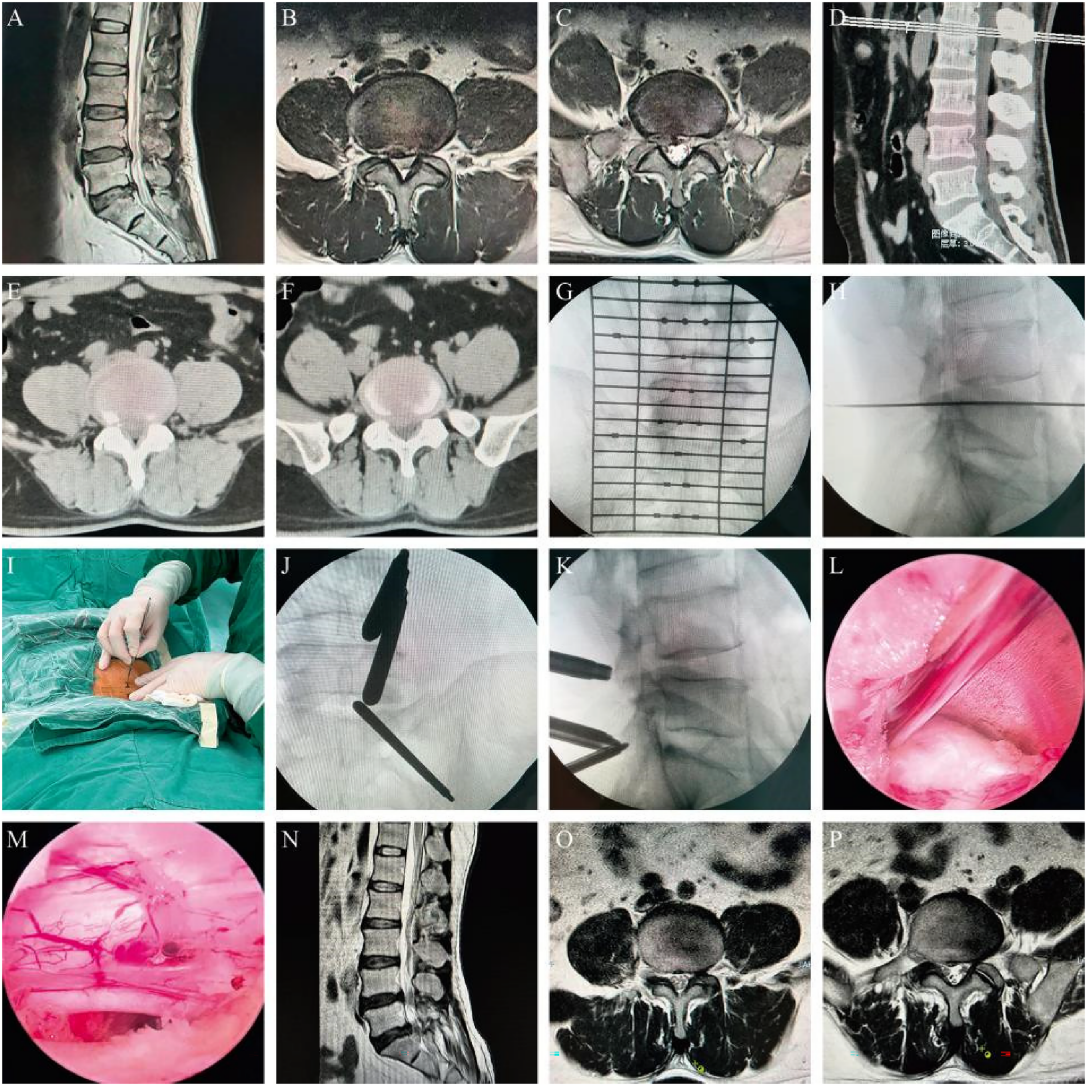
A 65-year-old male patient diagnosed with L4/5 and L5/S1 disc herniation who underwent UBED surgery. (**A**–**F**) Preoperative MRI and CT scans; (**G**,**H**) preoperative localization; (**I**) skin incision; (**J**,**K**) sheath located at the good position; (**L**) herniated nucleus pulposus at L4/5 segment; (**M**) sufficient decompression of the nerve root at the L4/5 segment; and (**N**–**P**) postoperative MRI scans showing satisfactory decompression.

Surgery was successfully performed on all geriatric patients, with a mean operation time of 46 min (range: 32–75 min). Patients were able to stand on their feet with a lumbar brace 2–6 h after postoperative bed rest. The mean duration of hospitalization was 3.4 ± 0.7 days. All patients were followed up for an average duration of 14.2 ± 1.9 months (range: 12–16 months). The leg pain VAS score decreased from 8.37 ± 1.21 preoperatively to 2.03 ± 0.61 immediately after surgery, 1.56 ± 0.32 at 1 month, 1.16 ± 0.45 at 6 months, and 0.91 ± 0.26 at 12 months postoperatively. Similarly, the ODI score also decreased from 61.21 ± 11.06 preoperatively to 27.52 ± 10.41 immediately after surgery, 19.12 ± 7.05 at 1 month, 12.17 ± 5.21 at 6 months, and 8.56 ± 4.32 at 12 months postoperatively. Statistically significant differences were observed in both VAS and ODI scores at each follow-up time point compared with preoperative parameters (Table 2, Figures 1, 2). There were 53 excellent cases, 12 good cases, and 7 fair cases based on the modified MacNab criteria at 12 months postoperatively, resulting in an excellent and good rate of 90.2%. Only three cases were found to be complicated with lower limb numbness, all of which were completely recovered via conservative treatment in 2–4 weeks. No infections or iatrogenic neurological deficits occurred in all patients. In addition, no patient reported recurrent disc herniation during the follow-up period. A typical case is presented in Figure 3.

**Table 2:** Leg pain VAS and ODI scores preoperatively and at each time point postoperatively.

	Preoperative	Immediately after surgery	Postoperative 1 month	Postoperative 6 months	Postoperative 12 months
Leg pain VAS	8.37 ± 1.21	2.03 ± 0.61*	1.56 ± 0.32*	1.16 ± 0.45*	0.91 ± 0.26*
ODI	61.21 ± 11.06	27.52 ± 10.41*	19.12 ± 7.05*	12.17 ± 5.21*	8.56 ± 4.32*

* *P* < 0.01, score at each time point postoperatively vs. preoperative postoperatively score.

## 4 Discussion

Symptomatic LDH is the most common lumbar degenerative disease, which causes low back pain and/or sciatica. Conservative treatment is usually preferred and often yields satisfactory results. With the aging population in China, the number of elderly patients with LDH is increasing. These patients often present with a long disease course, severe symptoms, and frequently have comorbidities such as coronary heart disease, hypertension, and diabetes. When conservative treatment proves ineffective, how to relieve pain through simple and effective surgery while improving the prognostic quality of life becomes a concern that deserves attention. Complex surgeries with extensive trauma increase the risk of perioperative complications for patients, and the incidence of adjacent vertebral diseases after intervertebral fusion surgery also significantly increases (21).

Nowadays, OLM has been considered the gold standard surgical treatment for symptomatic LDH (8, 22, 23). In recent years, minimally invasive spinal surgery techniques and endoscopic instruments have been continuously developed, as along with the increasing patient demand for reduced intraoperative injuries. PELD is widely used for symptomatic LDH. This technique has shown excellent results, with the advantage of greater preservation of bone, reduced soft tissue trauma, and faster recovery times (24, 25). However, the PELD technique has certain limitations, especially for beginners, as its operational flexibility is limited by the use of a single channel. At present, the UBED technique is gradually emerging as a treatment for lumbar degenerative diseases such as symptomatic LDH and lumbar spinal stenosis (16, 26). In addition, UBED has shown good therapeutic effects in the treatment of cervical and thoracic spinal diseases (27–29). We believe that UBED compensates for the operational flexibility of PELD while maintaining the advantages of minimally invasive techniques and enhanced visualization; for young physicians, the learning curve for UBED is relatively smooth. UBED is implemented using two independent channels on the unilateral side: one for the visualization and the other for working instruments. This separation of visualizing and working portals facilitates surgical operations compared to single-portal endoscopy, which is convenient for the extraction of protruded discs. It also provides a magnified and clear surgical field of vision while improving operational flexibility and helping the surgeon to conduct precise and complete decompression (16, 18).

Eun et al. performed nucleus pulposus removal on 11 patients with LDH using the UBED technique. After 14 months of follow-up, the results showed that the VAS score of leg pain decreased from 7.88 ± 1.24 preoperatively to 0.87 ± 0.64 postoperatively, and the ODI score decreased from 51.73 ± 18.57 preoperatively to 9.37 ± 4.83 postoperatively, indicating the effectiveness and safety of the UBED technique in treating LDH (30). Soliman (15) reported treating 43 cases of symptomatic LDH with the UBED technique. At the 24-month postoperative follow-up, 95% of patients expressed satisfaction with the therapeutic outcomes. In our study, statistically significant differences were observed in both VAS and ODI scores at each follow-up time point compared with preoperative parameters (Table 2, Figures 1, 2). There were 53 excellent cases, 12 good cases, and 7 fair cases based on the modified MacNab criteria at 12 months postoperatively, resulting in an excellent and good rate of 90.2%. A prospective study focused on 40 patients with single-segment LDH (31), and the researchers treated the patients using UBED and PELD techniques, respectively. After 6 months of follow-up, both groups showed significant reductions in postoperative leg pain VAS and ODI scores compared to preoperative parameters. However, the PELD group performed better in terms of intraoperative bleeding volume, surgical time, length of hospital stay, and short-term postoperative pain relief than the UBED group. Chang et al. (32) reported no significant difference in leg pain VAS or ODI scores at 12 months after surgery between the UBED and OLM groups. However, the UBED group showed an advantage in immediate postoperative back pain. A systematic review presented the complications associated with the UBED technique, such as incision or deep infections, iatrogenic nerve injuries, and dural sac tears (20). Only three cases were found to be complicated with lower limb numbness in our study, all of which were completely recovered via conservative treatment in 2–4 weeks. No infections or iatrogenic neurological deficits occurred in all patients.

Although the UBED learning curve is relatively flat, it still requires basic endoscopic operation skills. Based on our experience, we have some insights into this technique. First, how to quickly seek a safe and effective operating space? We usually reach the junction point between the upper vertebral plate and the root of the spinous process in the first step to establish a base area. Second, the hydrostatic pressure should be controlled to avoid intracranial pressure rise caused by high pressure. It is suggested that the hydrostatic pressure in lumbar surgery should be maintained at 25–30 mm Hg. Third, for fear of air blockage during operation, attention should be paid to removing bubbles in the brine flushing pipeline. Fourth, if the ligamentum flavum adheres to the dural sac, , only the superficial layer of ligamentum flavum can be stripped to avoid dural sac tear, leaving the adhesion area to maintain the integrity of the dural sac. This study has certain limitations. It was a single-center retrospective study with a relatively small sample size, and the varying duration of follow-up might have influenced the results.

## 5 Conclusion

We considered that UBED achieved satisfactory results and provided a minimally invasive, effective, and safe alternative for the treatment of symptomatic LDH in geriatric patients. In addition, a well-designed randomized controlled study with a large sample, multicenter data, and prolonged follow-up is required to verify the present results and draw a more convincing conclusion.

## Data Availability

The original contributions presented in the study are included in the article/Supplementary Material; further inquiries can be directed to the corresponding author.

## Geenrative AI statement

The author(s) declare that no Generative AI was used in the creation of this manuscript.
